# Chorea as the First Sign in a Patient with Elderly-Onset Systemic Lupus Erythematosus

**DOI:** 10.1155/2012/317082

**Published:** 2012-02-13

**Authors:** Yuko Ariizumi, Tetsutaro Ozawa, Takayoshi Tokutake, Izumi Kawachi, Masaki Hirose, Shinichi Katada, Shuichi Igarashi, Keiko Tanaka, Masatoyo Nishizawa

**Affiliations:** ^1^Department of Neurology, Brain Research Institute Niigata University, Niigata 951-8585, Japan; ^2^Department of Neurology, Kanazawa Medical University, Ishikawa 920-0293, Japan

## Abstract

The case of an elderly patient who had chorea as an initial symptom of systemic lupus erythematosus (SLE) accompanied by antiphospholipid syndrome (APS) is reported. A 68-year-old woman suddenly developed chorea of her left arm and leg. Magnetic resonance imaging (MRI) of the brain demonstrated a focal lesion in the right caudate head, which showed hyperintensity on fluid-attenuated inversion recovery and diffusion-weighted imaging. This condition was thought to be a common form of vascular chorea, which is likely to occur in elderly individuals; however, the laboratory data of this patient finally fulfilled the diagnostic criteria of SLE and APS. Physicians should be careful in diagnosing elderly individuals simply as having a vascular chorea because this symptom can be the initial manifestation of SLE or APS.

## 1. Introduction

Chorea rarely occurs in young patients with systemic lupus erythematosus (SLE) or antiphospholipid syndrome (APS) [1, 2]. However, the incidence of chorea in elderly patients with SLE or APS has not been established. In young patients, chorea has been reported to be presenting clinical feature of SLE or APS [2], but whether it can be an early sign of elderly-onset SLE or APS remains to be elucidated. Here, we report a patient with elderly-onset SLE who had chorea as an initial symptom of SLE accompanied by APS. This condition could be confused with a common form of vascular chorea [3], which is likely to occur in elderly individuals. This paper provides information useful to make diagnosis on a rare case of elderly-onset SLE whose initial symptom is chorea.

## 2. Case Presentation

A 68-year-old woman suddenly developed involuntary movements of her left arm and leg. The symptom deteriorated rapidly. She was admitted to our hospital two weeks after symptom onset. The patient had papillary thyroid cancer at the age of 67 years. Her medical history included no psychotropic medications or head trauma. There was no family history of neurological diseases.

**Figure 1:**
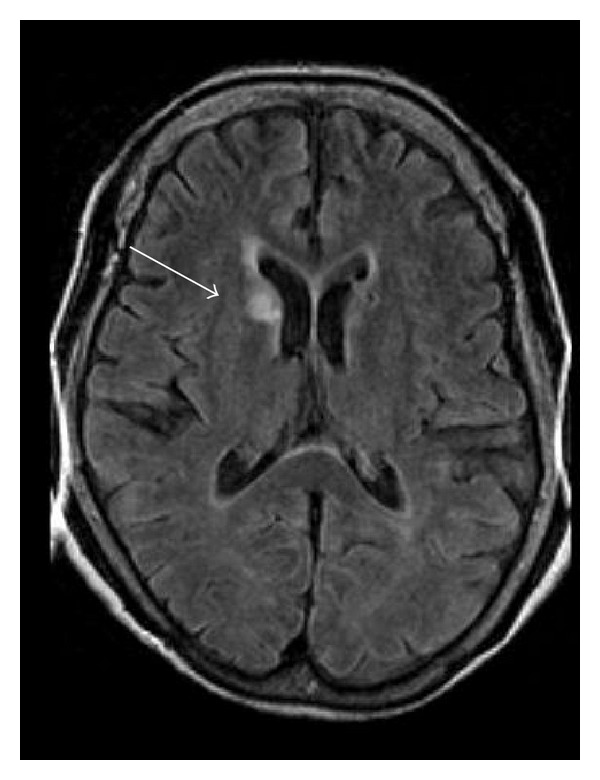
Magnetic resonance imaging of the brain shows a focal lesion in the right caudate head, which shows hyperintensity on fluid-attenuated inversion recovery imaging.

On examination, irregular, seemingly random, semi-directed movements of the left upper and lower extremities were seen. The patient also showed a scowling face and darting tongue. These choreic movements substantially disabled the patient during wakefulness, but they disappeared during sleep. Her muscle tone and tendon reflexes were normal, and no pathological reflexes were observed. Magnetic resonance imaging (MRI) of the brain demonstrated a focal lesion in the right caudate head, which showed hyperintensity on fluid-attenuated inversion recovery (Figure 1) and diffusion-weighted imaging. This lesion showed hypointensity on apparent diffusion coefficient mapping, and it was slightly contrast-enhanced with gadolinium. These findings on MRI indicated an ischemic stroke in the caudate head; however, laboratory findings disclosed that this patient's disease process was not that simple. The blood cell count showed a mild leucopenia. There were high titers of antinuclear antibody, anti-DNA antibody, and anti-SSA antibody on two occasions 12 weeks apart. We diagnosed her as having neuropsychiatric syndromes of SLE (NPSLE), according to the American College of Rheumatology criteria [4], and subsequent criteria for NPSLE [5]. Furthermore, the patient also had lupus anticoagulant on two occasions 12 weeks apart, suggesting that this patient also had APS. Cerebrospinal fluid analysis was unremarkable. In coagulation examination, the levels of fibrin degradation product, D-dimer, and thrombin-antithrombin complex were elevated slightly. 

The patient's chorea improved spontaneously two weeks after admission. Haloperidol (1.5 mg/day) further ameliorated the symptom. We also started antiplatelet therapy using aspirin, which also has an inhibitory effect on endothelial activation and inflammation [6]. Brain MRI performed 2 months after disease onset showed disappearance of the caudate lesion, in keeping with the improvement of the chorea. Steroid therapy was started 3 months after disease onset, and no relapse of chorea has been seen in the patient. The patient gave her informed consent before the evaluations and treatment were performed. 

## 3. Discussion

Diagnosis of this elderly patient as having NPSLE accompanied by APS was puzzling because she suddenly exhibited chorea caused by a focal brain lesion that could be confused with a common form of vascular chorea [3]. Of note, MRI clearly demonstrated the focal brain lesion causative of chorea in this patient despite the fact that MRI cannot detect the causative brain lesions in most patients with chorea associated with SLE or APS [7, 8]. Thus, it was difficult to presume that SLE or APS underlay chorea in this elderly patient. This issue is of concern to the treatment of elderly patients with chorea as pharmacological intervention for chorea may vary according to the underlying condition such as SLE or APS. 

 The MRI findings indicate that our patient had a cerebral vasculopathy in the basal ganglia, which is associated with development of the neuropsychiatric event such as chorea. The relationship between autoantibodies and neuropsychiatric events of NPSLE should be considered with regard to the clinical manifestation in this patient. Recently, antiribosomal P and antiphospholipid antibodies have been reported to be associated with neuropsychiatric events, which occurred around the time of diagnosis of NPSLE [9]. The presence of antiphospholipid antibody mainly contributes to chorea and cerebrovascular diseases associated with thrombotic events in patients with NPSLE [9, 10]. However, in most patients with NPSLE, the absence of lesions on MRI [7, 8] argued against chorea as a direct consequence of large thrombotic events associated with antiphospholipid antibody. The pathogenic findings in NPSLE patients showed that small vessels in the central nervous system were disrupted with fibrin thrombi or vasculitic changes, and depositions of immune complexes involving antiphospholipid antibody were seen [11]. Therefore, small vessel vasculopathy in the basal ganglia should be considered as a cause of chorea associated with NPSLE accompanied by APS. In our patient, the damage of small vessels might become still worse, as aging factors influence vulnerability of small vessels. Taken together, these observations suggest that our patient with elderly-onset NPSLE was highly susceptible to small vessel vasculopathy, which led to the consequence that the caudate lesion appeared on MRI, and chorea occurred as the first sign of the disease. 

 The prevalence of SLE or APS among patients with chorea caused by cerebral vasculopathy has not been determined. Furthermore, the incidence of this type of chorea occurring as the initial symptom in elderly patients with SLE or APS remains to be elucidated. Physicians should be careful in diagnosing elderly individuals simply as having a vascular chorea because this symptom can be the initial manifestation of SLE or APS.
